# Myelitis and Radiculitis Complicating Varicella-Zoster Virus Reactivation

**DOI:** 10.1590/0037-8682-0290-2020

**Published:** 2020-11-13

**Authors:** Diogo Goulart Corrêa, Niedja Santos Gonçalves Tsuno, Luiz Celso Hygino da Cruz

**Affiliations:** 1 Clínica de Diagnóstico por Imagem/DASA, Departamento de Radiologia, Rio de Janeiro, RJ, Brasil.; 2 Universidade Federal Fluminense, Departamento de Radiologia, Niterói, RJ, Brasil.; 3 Laboratório Exame/DASA, Departamento de Radiologia, Brasília, DF, Brasil.

**FIGURE 1:**
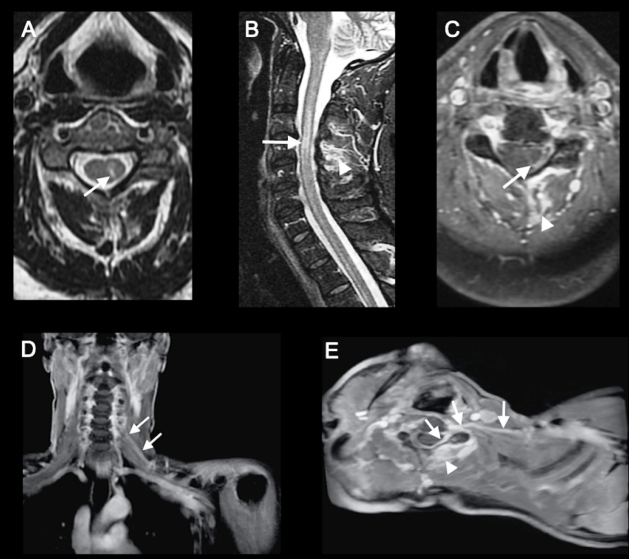
The reactivation of varicella zoster virus is complicated by myelitis and radiculitis. Cervical spine MRI showing hyperintense signal in the left gray matter columns of the spinal cord at C3 to C5 based on axial T2-weighted imaging (**arrow in A**) and sagittal STIR (**arrow in B**), and with contrast enhancement (**arrow in C**). Contrast enhancement was also detected in the left C5 spinal nerve root and the nerve (**arrows in D and E**), suggesting myelorradiculitis. The edema and contrast-enhancement in the corresponding homolateral paravertebral muscles (arrowheads) are of additional note.

A 44-year-old man presented with small painful vesicular rash on his left shoulder and arm. After three days, the patient progressively lost motor strength and sensitivity in his left upper limb. Magnetic resonance imaging (MRI) showed a hyperintense signal in the left gray matter columns of the cervical spinal cord at the level of C3 to C5 based on T2-weighted and short tau inversion recovery (STIR) images, with slight contrast-enhancement. MRI also demonstrated contrast-enhancement in the left C5 nerve root and the adjacent homolateral paravertebral muscles, and this was associated with edema (Figure 1A-E). Analysis of cerebrospinal fluid (CSF) demonstrated elevated white blood cell counts with 100% mononuclear cells and a positive polymerase chain reaction for varicella-zoster virus (VZV). The patient was treated with intravenous acyclovir and a pulse of intravenous methylprednisolone.

Primary infection with VZV in susceptible individuals causes varicella, which usually is harmless in healthy patients. The virus subsequently enters a phase of latency in ganglionic neurons. On reactivation, which is usually triggered by a decline in specific cell-mediated immunity, the virus often spreads peripherally causing herpes zoster. However, it can also spread centrally to cause neurological complications, including myelitis^1^ ^,^ ^2^. The pathogenesis of VZV-related myelitis is postulated to be related to direct viral invasion. Vascular and autoimmune mechanisms have also been proposed in its pathophysiology^3^. 

Although not specific, the unilateral involvement of gray matter in the spinal cord is one form in which VZV myelitis presents. It might also present itself as transverse myelitis or Brown-Séquard syndrome. Thus, VZV myelitis is diagnosed through clinical suspicion and subsequent virologic confirmation^1^ ^,^ ^2^.
